# Sociodemographic Influence on the Management of Orofacial Cleft in Urban and Rural Indonesia

**DOI:** 10.1177/10556656241288762

**Published:** 2024-10-15

**Authors:** H. Hasanuddin, Aisha AH. Al-Jamaei, M. Ruslin, Fridus Steijlen, A. Tajrin, M. N. Helder, T. Forouzanfar

**Affiliations:** 1 Department of Oral and Maxillofacial Surgery, 1209 Amsterdam UMC, Amsterdam, The Netherlands; 2 Department of Oral and Maxillofacial Surgery, 4501 Leiden University Medical Center, Leiden, The Netherlands; 3 Department of Dental Public Health and Preventive Dentistry, Faculty of Dentistry, 64739 Hasanuddin University, Makassar, Indonesia; 4 Department of Oral Medicine, Periodontology, and Radiology, Faculty of Dentistry, 108522 Sanaá University, Sanaa, Yemen; 5 Department of Oral Surgery and Oral Medicine, Collage of Dentistry, 516998 Al-Razi University, Sana’a, Sanaa, Yemen; 6 Department of Oral and Maxillofacial Surgery, 355664 Faculty of Dentistry, 64739 Hasanuddin University Indonesia, Makassar, Indonesia; 7 Department of Social and Cultural Anthropology, Faculty of Social Science, 1190 Vrije Universiteit Amsterdam, Amsterdam, The Netherlands

**Keywords:** sociodemographic factors, orofacial cleft, patient care, urban-rural, Indonesia.

## Abstract

**Objective:**

To explore the social experiences of orofacial cleft patients and contextualize sociodemographic influence on management disparities in rural and urban areas of Indonesia.

**Design:**

This study explores patients’ lives in two demographical settings in South Sulawesi Province, Makassar and Selayar Islands Regency. It employs ethnography, including open-ended interviews and observations of patients, their neighborhoods, schools, and workplaces. Secondary data from the two regencies and South Sulawesi province are employed to demonstrate demographic disparities in patient care between urban and rural areas. Thematic content analysis was performed according to socio-demographical differences and networks involved in the management.

**Participants:**

This study engaged a diverse group of participants, including patients, family caregivers, medical team members, and patient peers (n = 40), primarily from middle to low-income families.

**Results:**

Parents in both regions had limited knowledge about treatment modalities, which resulted in concerns regarding the safety of surgical procedures and the postponement of mandatory surgeries. In rural areas, patients faced significant financial burdens when referred to Makassar. In contrast to parents in Selayar, parents in Makassar prioritized more social interaction to ensure that their children attended school, college, and work. Patients in both settings reported facing various obstacles at different stages of their lives.

**Conclusions:**

Sociodemographic conditions contribute to disparities in treatment, social positioning, and self-perception. Promoting education about the safety of medical and rural surgical procedures is vital. Involving patients in public activities and providing support from family caregivers is paramount to nurturing patients’ optimistic outlooks.

## Introduction

Cleft lip and/or cleft palate (CL/P) are common visible congenital disabilities that affect one out of every 750 births worldwide.^1^ In South Sulawesi-Indonesia 2018, Basic Health Research reported 454 babies born with CL/P.^2^ Patients worldwide have been asserted to experience minor psychological and social problems.^3–5^ The social problems may include negative stigma, livelihood, and employment issues, such as workplace stigma, job discrimination, and comments or jokes behind their backs.^6^ These societal problems require inclusive management involving medical treatments and family and community caregivers’ support. Unfortunately, due to financial constraints, misunderstandings about the causes, lack of medical specialists, and access to referral hospitals, patients often fail to receive comprehensive biomedical treatments and necessary support. Further, demographical conditions exacerbate the effective delivery of medical treatments and necessary supports. Hence, it is crucial to understand the patient's sociodemographic context to comprehend challenges better and develop evidence-based strategies to address them.

Several reports discuss the healthcare disparities between urban and rural areas, especially in primary healthcare centers.^2,7,8^ This is particularly true in Southeast Asian countries, a region of enormous social, economic, and political diversity.^7^ Indonesia, situated in Southeast Asia, is an archipelago of social diversity, including highly variable demographics, economic backgrounds, and education. In 2004, centralized one-size-fits-all healthcare insurance was launched under Indonesia's National Healthcare Program (JKN) to bridge the diversity gap between rural and urban areas. However, the approach seemed not to address the complexity and diversity in population density and dispersion across islands, health beliefs, human development, and community discrepancies. The decentralization of governance to 354 districts in 2001 and 514 districts further increased health system heterogeneity and exacerbated equity gaps.^9^

Previous research has highlighted the challenges of managing illness in rural areas of low- to middle-income countries.^10^ It revealed that access to adequate health facilities remains challenging, and comprehensive treatments are not readily available. This situation is worsened by individuals with lower socioeconomic status being more prone to CL/P.^10^ However, the study did not delve into the micro-level of social, economic, and demographic factors associated with the delivery of medical treatments.

Our research goes beyond the sociodemographic influence on medical treatments of CL/P. We studied two regions in South Sulawesi, Makassar and Selayar, each with unique social and demographic characteristics. Makassar is an urban area and the center for health services in South Sulawesi, while Selayar is a more rural region separated from the Sulawesi mainland. We analyzed how medical treatment was delivered and how patients were cared for within their communities. Our findings describe disparities in managing CL/P patients between rural and urban areas in Eastern Indonesia, using these regions as a detailed case study of the country's archipelagic condition.

Contextualizing the discrepancy of CL/P treatments between rural and urban areas, particularly in the case of Indonesia, a low-mid income and archipelagic country, contributes to a better understanding of challenges to developing evidence-based strategies to address them. Systematically identifying the disparities in CL/P treatment between rural and urban areas will help policymakers and healthcare professionals understand the problem's extent and prioritize interventions. Understanding barriers contributing to the discrepancy in treatments is crucial for designing effective interventions that address the root causes of the problem. Developing context-specific solutions is expected to bridge the gap in CL/P management and promote equitable access to healthcare services.

The terms “rural” and “urban” in this research specifically refer to how structures in these two settings, including health facilities, medical specialists, families, and communities, differ in providing patient care and assistance. Thus, this research proposes three research questions: 1) What socio-demographic conditions characterize Makassar as an urban area and Selayar as a rural area? 2) How do these socio-demographic conditions influence the management and patients’ social lives? 3) How have these challenges been addressed?

## Methods

### Study Design

This study used an ethnography approach to thoroughly understand the social experiences of CL/P patients in South Sulawesi between 2018 and 2021. The first author (HH), a CL/P social worker, a trained anthropologist, and a Ph.D. student, participated in the ethnography, and two other research assistants (NE and CDS) who have been working at Hasanuddin University's faculty of dentistry for the past two years conducted the interviews. They had previously been informed of the research's main objectives and data collection methods, specifically interviews.

This study combines three data series: secondary data on socio-demographic differences, open-ended interviews, and observations to ensure diverse representation. Secondary data contextualized the socio-demographic discrepancies between rural and urban areas. Interviews with patients, family caregivers, and medical teams were conducted to understand the disparity in the management of cleft between urban and rural areas, especially in capturing experiences and perspectives of individuals directly involved in cleft management, such as patients, family caregivers, and medical teams, to share their experiences, challenges, and perspectives. The observation was performed to see how the patient's social life differs in two settings.

Before data collection, the Faculty of Medicine, Hasanuddin University's Health Research and Ethics Committee, provided ethical clearance (No.UH14060319). All participants provided written informed consent before taking part in the study. This included informing participants about the study's rationale, potential benefits and risks, procedures, and the entire participation process, which was entirely voluntary and would have no consequences if they withdrew. This study followed the principles outlined in the Helsinki Declaration.

### Participants’ Inclusion and Exclusion Criteria

The study included a diverse group of participants (n = 40) living in the urban and rural regions of South Sulawesi Province, Indonesia: 1) patients with CL/P from middle to low-income families, 2) family caregivers of CL/P patients, 3) medical team members involved in CL/P patient care, and 4) patient peers. Patients were mainly identified and recruited by the medical team at the Celebes Cleft Center (CCC) Foundation. This team provided background information on the patients and shared their experiences working with individuals in the South Sulawesi regions.

Before collecting data, the researchers obtained informed consent from all informants. Patients were recruited from the Makassar and Selayar Islands Regency regions, as the study aimed to explore differences in patient experiences and access to care between urban and rural settings. Patients from the Selayar Islands Regency were explicitly included to gather data on the challenges faced by those living in more remote, rural areas, as the CCC, Hasanuddin University Dental Hospital, and Dentistry Faculty could not provide services in this region. The exclusion criteria were patients living outside of the targeted areas of Makassar and Selayar Islands Regency and those from high-income backgrounds.

### Stepping into the Fields

The researcher first informed the medical team in Makassar about the research plan and requested preliminary data. The medical team provided background information on patients and their experiences. The researcher (HH) contacted the dean of the Dentistry Faculty at Hasanuddin University and the head of the CCC medical team to gather additional information. The dean (MR) introduced two research assistants - NE and CDS - who had experience assisting research projects. They introduced the researcher to four patients and families in Makassar and helped with videotaping. After acquiring information about eligible patients and obtaining preliminary data in Makassar, the researcher (HH) approached gatekeepers in Selayar to introduce the researcher to eligible patients and their network to be included in the study.

### Positionality

Initially, the researchers were worried about being affiliated with the CCC but found that patients and families were more responsive as they knew the researcher was part of the CCC. The researcher explained that the purpose of the study was to gather information to encourage support and better treatment for patients. The researcher approached the study with an emic perspective to understand firsthand how patients, families, and the community view and treat CL/P.

### Data Analysis and Samples Included

Narrative data from interview transcripts and field notes were used to generate descriptive qualitative data. Thematic content analysis regarding health equity discrepancy was performed according to R. Mbau et al.'s 2023.^11^ R. Mbau et al. conducted a systematic review including 131 articles to assess the efficiency of health systems across the globe. Their study summarized factors that influence health system efficiency, including demographic and socioeconomic characteristics of the population, macroeconomic characteristics of national and sub-national regions, population health and well-being, governance and political aspects of these regions, and health system characteristics. As a result, those factors are regarded as a comprehensive variable that defines the sociodemographic context of a community associated with the health system. Our study used these characteristics to describe and compare the current sociodemographic conditions in urban and rural areas in eastern Indonesia.

The fieldwork data on demographic influences, socio-economic factors, and the availability of health services, hospitals, medical teams, and distance to referral hospitals was analyzed using an inductive coding approach (Table 1). Rather than relying on predetermined theories or frameworks, the codes and themes emerged directly from the data. This approach allowed the researcher to explore and understand the phenomenon without imposing predefined categories. The researcher utilized open-ended interviewing techniques to facilitate this inductive process, allowing participants to share their life stories and experiences without being constrained by any pre-existing categorizations. This openness enabled the researcher to capture the nuances and perspectives of the participants fully. After all, ATLAS.ti.8 was used to analyze narrative data.

**Table 1.** Overview of Themes and Most Important Findings.

Dimension	Urban Context	Rural Context
*Demographic influence on management*	Despite the untimely administration of treatments, every patient received care. Time savings on travel to a referral hospital Patients have access to cleft foundations that offer complementary surgical interventions.	More patients with untreated clefts Extended travel time to the referral hospital and increased financial repercussions.
*Socio-economic influence on management*	The lack of parental knowledge regarding treatment modalities Postponing treatments Concerns regarding the safety of surgical procedures. Patients who are not treated comprehensively and promptly may experience social difficulties. Patients pursue Bachelor's or High School Diploma Did not give palate surgery, considering it is not seen and patients did not have a problem. Adult patients make more effort to seek follow-up treatments and therapy. The government has better collaboration with the Cleft Foundation.	The lack of parental knowledge regarding treatment modalities Postponing treatments Concerns regarding the safety of surgical procedures. Initially, parents refuse treatment, considering cleft as God's given. Patients quit elementary school or have no education. Did not give palate surgery, considering it is not seen and patients did not have a problem. Adult patients have less effort to seek follow-up treatments and therapy. The local government does not collaborate with the cleft foundation, so surgical missions have not been done in the area.
*Health services availability influences management.* Hospital and medical Team, distance to referral Hospitals.	Early detection, soon after the baby born. Medical team to do surgery and therapy are available. More comprehensive treatments, such as prostheses (speech bulb) and orthodontic treatments.	Did not detect the cleft earlier. Medical team to do surgery and therapy is not available. Patients must be referred to Makassar for surgical treatments.

In Makassar, we included 25 individuals in our study. The group consisted of four patients, five parents, three peers of patients, four extended family members, two patients’ workmates, and seven medical teams. These medical teams comprised three oral and maxillofacial surgeons, a prosthodontist, a speech therapist, and two dentists. In Selayar, our study group consisted of twenty individuals: five patients, four medical teams, four parents, five peers of patients, and two patients’ siblings. We gathered information on the patient's exposure to treatments, current CL/P conditions, treatments undertaken, and responses to the current therapies.

We delved into publicly available statistical data to give an overview of sociodemographic and economic differences between urban and rural areas. The data demonstrates demographic differences in patient care in urban and rural areas, including the Indonesian Central Bureau of Statistics. Data refers to secondary data based on Makassar's, Selayar's, and South Sulawesi Province's Health Profiles as reported in 2021.^12–14^

### Rural and Urban in the Context of our Research

Bennet et al. highlight the need to clearly define rural areas as challenging to monitor public health.^15^ To ensure accurate results, it's important to define ‘rural’ for each study. Our study defined rural and urban areas based on population density, distance from referral hospitals, socioeconomic factors, and health system characteristics, following Bennet et al.'s definitions. This aligns with Mbau et al.'s review, which identified variables affecting health system efficiency, including macroeconomic, regional, population health, and governance factors.^11^ In line with this, our research focused on investigating and comparing public health indicators in urban and rural areas, specifically in Makassar, the urban capital of South Sulawesi, and Selayar, a rural regency consisting of 130 islands separated from the mainland of Sulawesi, Indonesia.

## Findings

### Sociodemographic Difference Between Rural and Urban Areas

**Figure 1.**
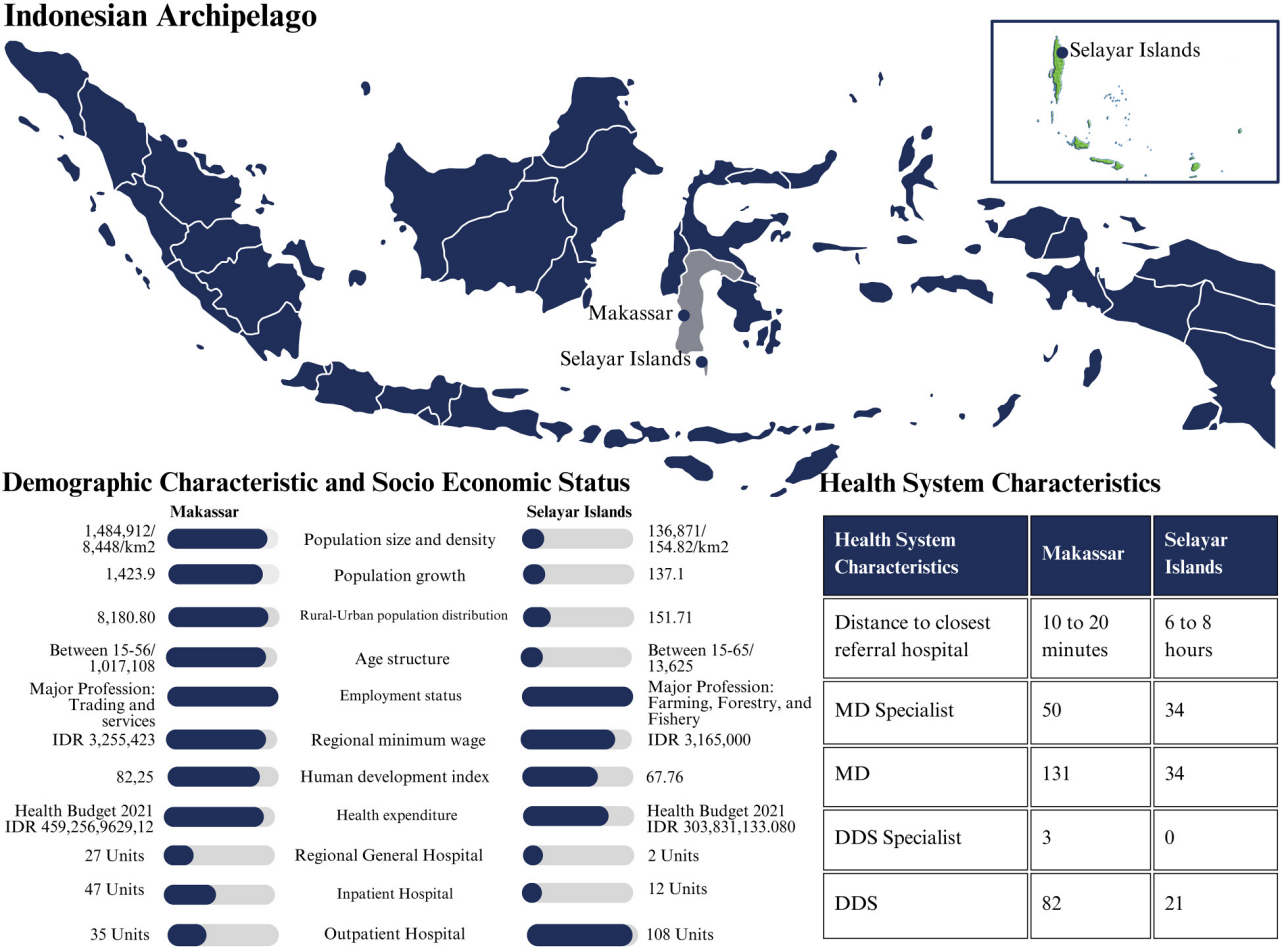
Health system comparison between regions based on R. Mbau et al. category.^16^.

The disparities in primary professions, income distribution, and human development index between Makassar and Selayar had far-reaching consequences for the health systems in these cities (Figure 1). Makassar's emphasis on trade and services, its higher average income, and its higher human development index showed superior access to healthcare, infrastructure, and resources. Additionally, Makassar had a sophisticated healthcare system, including a more comprehensive array of specialized services, better-equipped facilities, and a substantial healthcare workforce. In contrast, Selayar mainly depends on agriculture, forestry, and fishing, with its lower average income and human development index. It is characterized by a shortage of healthcare facilities, a need for medical professionals, and geographical constraints that make healthcare facilities challenging to access.

Makassar, with a larger population and higher population density, showed a great need for healthcare services such as primary care, specialized care, and emergency care (Figure 1). In contrast, Selayar, with a smaller population and lower population density, demonstrated a lower demand for such healthcare services. However, considering the rural-urban population distribution, it was clear that people in Selayar faced challenges in accessing healthcare facilities and services, particularly in more remote areas (Figure 1).

### Health System Characteristics

Makassar had more beneficial health care than Selayar (Figure 1). Makassar had a more significant health budget, suggesting more healthcare facilities and resources, including regional general hospitals, community health centers/inpatient hospitals, and community health centers/outpatient hospitals. This made healthcare more accessible—a 10–20-min drive to the nearest referral hospital. Makassar had more medical professionals, including 50 MD specialties, 131 MDs, and 82 DDS.

Selayar's healthcare system was challenging. Selayar had a smaller health budget, alleviating healthcare resources (Figure 1). The nearest reference hospital was a 6-to-8-h drive, including a 2.5-h ferry trip, due to Selayar's archipelago's geography. This hampered emergency care. Selayar also had fewer hospitals than Makassar: two regional general hospitals, twelve community health centers/inpatient hospitals, and 108 community health centers/outpatient hospitals. The region needed additional medical personnel.

### Health System Disparities Affect Patient Management and Social Life at the Micro Level

#### Socio-Demographic Influence on Management

In rural regions, socioeconomic conditions influenced cleft management. Lack of surgery and therapy information delayed parents seeking treatments for their children. Surgical safety concern influenced their treatment decisions. In contrast, urban parents had higher educational backgrounds, many holding bachelor's degrees or High School Diplomas (Figure 1). Due to the increased Government-Cleft Foundation collaboration, cleft patients could get financial assistance and specialist treatments. Urban adult cleft patients sought follow-up treatment more frequently. The urban environment ensured that all patients received treatment, even if treatment was delayed, benefiting from shorter hospital travel times and improving their overall treatment:

“*Most people in my neighborhood know I was born with a cleft lip and palate. My parents used to take me out to meet our neighbors and attend parties or family events. I was lucky to get lip surgery when I was three months old. Although I did not get palate surgery earlier, lip surgery gives me a better appearance. I know two patients in my neighborhood who did not get surgery and spent most of their time at home. The first one, a child, died when he was five. Another one was an adult. He had returned to his village, but we do not know where.”* [Quote from a patient in Makassar]

Socioeconomic factors affected rural cleft treatment comparable to urban. Rural parents delayed treatment due to a lack of treatment knowledge (Table 1). Many rural parents did not attend elementary school. This low educational background may influence cleft care knowledge and decision-making. Some parents believed cleft was a God-given, resulting in the postponement of treatment:

“*We Muslims must accept what Allah has given us. Of course, I was shocked to see my daughter's condition. I asked myself why she had this kind of deformity. Again, I speak to myself; it is Allah's given. Other kids used to mock her when she was a kid by imitating how she talked, but her mother always advised her to be patient and remember that Allah's given. He would show us how to fix it; just be patient.* [Quote from a Patient's father in Makassar]

Rural adult cleft patients seek follow-up treatments less often due to inadequate healthcare resources and a need for a better understanding of comprehensive care. Therefore, a rural government-Cleft Foundation collaboration to do CL/P surgical missions was necessary. There are more untreated patients in rural areas, considering travel time and expensive living costs in urban referral hospitals.

Patient social life experiences in Makassar and Selayar differed due to parental attitudes toward patients’ educational attainment, hope, support, and exposure to diverse social situations and life experiences (Table 1). Makassar parents often encourage their children to achieve their objectives and socialize despite their disabilities. This helped patients stay cheerful and manage their medical conditions.

“*She has reasonable confidence and makes many friends. She is also lucky to have a supportive brother. Although they are living away, they always speak to her by phone. She likes to spend her leisure time with her friends or our neighbors. Her friends often mocked her when she was a kid, especially in elementary school, but her mother always reinforced that her friends were just kids. They did not know what they were doing or realize what they said was hurting them. We always are all ears.”* [ Quote from a Patient's father in Makassar]

Makassar's parents believed social interaction was feasible despite their child's limitations. They constantly reassured their children that they could succeed. This helped people remain optimistic and manage their CL/P. Selayar parents worried about their children's social and academic coping skills. When a patient plans to go to college, their relatives worry about the patient's social and academic coping skills. Another patient's mother told his son when he asked for palate surgery: “*Do not worry, although you do not get palate surgery and have a speech problem, getting a wife will not be difficult*.”

The parents and family of the cleft palate patient were unaware of his cleft palate at birth. They realized it after his mother breastfed him, and his nose dripped milk when he was three months old. His parents then took him to Selayar General Hospital to consult with a medical doctor who advised a palate surgery. Because no surgeons were in the regional hospital, he was advised to refer the patient to Makassar and explain the procedures. However, after the explanation, his parents made their presupposition that if the surgery failed, he would not speak, eat, or drink. They kept postponing surgery because their son did not report personal or societal problems.

#### Health Services Availability

In an urban setting, access to healthcare facilities and skilled medical specialists increases the likelihood of early cleft identification after birth. Furthermore, urban hospitals and healthcare facilities performed surgery and complete treatments, including prostheses (speech bulbs) and orthodontics (Table 1). These conditions enabled urban patients with cleft lip and palate to receive thorough medical attention and achieve improved long-term outcomes. This included having access to a team of specialists who collaborate to create personalized treatment plans and engaging with the community to assist patients and their families in dealing with emotional and social difficulties. Given the multitude of factors involved, individuals with cleft conditions have a greater chance of enhancing their overall quality of life.

Limited health services availability in rural areas has negatively affected cleft management. In Selayar, there was no cleft center. Two specialists to provide surgical treatments (oral and maxillofacial surgery and plastic surgery) were unavailable, not to mention other specialists to provide patients with more comprehensive treatments and therapies. The Cleft Foundation did not do charity work to give free surgery. Hence, when the parents consulted the local medical team about their children's condition, the medical team would advise or refer them to Makassar to get surgical treatments and therapy. Those challenges led to delays in the early identification and management of CL/P patients, explaining the high number of untreated CL/P cases in Selayar. These hindered timely and necessary treatments, creating barriers to comprehensive care.

## Discussion

Grimes et al. conducted a systematic review to identify the dimensions and barriers to delivering surgical treatments in low—and middle-income countries, which include cultural, financial, and structural barriers.^17^ Our current study further contextualizes Grimes et al.'s dimensions and barriers regarding healthcare access and management disparities between urban and rural areas of middle-income countries. Urban areas like Makassar demonstrated better access to healthcare services, well-equipped facilities, and a larger healthcare workforce. In contrast, rural areas like Selayar faced challenges in accessing healthcare due to geographical constraints and limited resources.

This study showed that urban parents of cleft patients with higher levels of education had a favorable impact on their treatment-seeking behaviors, the quality of therapy received, their social position, access to specialized treatments, and financial assistance. In rural settings, however, several factors contributed to the delay of medical treatment for cleft patients. These included parents’ lower levels of education, inadequate understanding of comprehensive treatment, limited community exposure of the patients, and misunderstandings that cleft conditions were God-given. Such disparities identified a call for targeted policies and interventions to improve access to healthcare in rural areas, establish specialized cleft centers, and increase the number of medical professionals. These include government-cleft foundation collaborations, surgical missions in rural areas, and efforts to provide free surgeries. Addressing access barriers, promoting education, and raising awareness aim to enhance CL/P management and ensure comprehensive care for all individuals affected by this condition.

A key difference between the social life experiences of patients in Makassar and Selayar lies in the parental attitudes towards educational achievement, hope, support, and exposure to various social conditions and life experiences. Parental approval played a pivotal role in their children's accomplishments. Parents in Makassar believed social interaction was possible despite their children's limitations and consistently reassured their children that they could achieve their goals. This fostered a positive outlook, helping patients manage their conditions. On the contrary, parents in Selayar appeared to set lower expectations for their children's social and academic achievements. It was observed that parents were worried about their children's ability to navigate social and educational challenges, expressing concerns about their coping skills. The families were particularly anxious about their children's ability to adapt to new social circles and individuals when attending college. Overall, parents in Selayar were uncertain about their children's ability to adjust to new environments.

We found that patients of different ages and educational backgrounds encountered unique challenges, necessitating tailored support. This support should extend beyond the medical team or parents and include the patient's entire social circle, given its impact on their worldview and quality of life. In Selayar and Makassar, patients reported instances of bullying, particularly in their early years, such as elementary school. Their speech became a source of mockery, leading to social exclusion and misunderstandings. Although bullying subsides after elementary school, patients grapple with self-esteem issues and academic struggles when articulating questions or ideas. Adult patients often feel insecure about public speaking. These experiences illustrate how stigma can personally affect a patient's self-image, making them feel different and insecure about presenting themselves in public.

The current research indicated that in Makassar and Selayar, lip surgeries were performed for patients. However, due to the misconception that invisible cleft palates would not cause any issues, most parents refrained from timely cleft palate surgeries for their children. In Selayar, parents expressed their concerns about possible risks associated with surgical procedures, leading to further delays in treatment. Over time, as children grow, parents notice that untreated cleft palates result in speech difficulties and social challenges. When parents recognized these problems and opted for surgery, it was often too late for cleft surgery and speech therapy to fully address the speech issues. Interestingly, this delay often corresponded with the development of the child's ability to adapt to their condition. This led parents to mistakenly assume that their child did not face any difficulties in daily activities and did not require surgery or therapy. The parent's perspective was that their child could adapt even with untreated clefts, eliminating the need for surgical intervention and treatment. This finding resonates with the study by Nilsen and Anderson on patients suffering from non-malignant chronic pain.^18^ Their analysis revealed that patients with conditions like chronic pain strive to lead a normal life after grappling with their situation. They found that patients are often forced to develop coping strategies due to their health condition and social environment, especially when they cannot access complete treatments from medical specialists for various reasons. When coping mechanisms emerge, people close to the patient often wrongly assume that the patient no longer needs treatment or therapy.

To solve the economic burden in management, in 2014, Indonesia's Ministry of Health announced that CL/P treatment would be covered by health insurance according to national regulations.^19^ However, demographic conditions also challenged the implementation of universal health coverage in Indonesia. 270.20 million people populate Indonesia, spread over 16.766 islands and 26.50 million low-income societies.^20^ This demographical condition had more financial consequences for building and implementing health services evenly distributed throughout Indonesia. CL/P patients often require surgeries performed by multidisciplinary medical specialists, therapies, and psychological support from birth to adulthood, making their management challenging. Our study reported that these facilities were often readily available in urban environments such as Makassar. Still, they were scarce in rural areas, leading to a remarkable disparity in the management of cleft patients between both settings. Addressing these disparities requires tailored strategies, including improving healthcare infrastructure, increasing the healthcare workforce in rural areas, and implementing targeted health promotion and prevention programs to address the specific needs of both urban and rural populations in Makassar and Selayar. Doing an ethnography study, given Eastern Indonesia's sociodemographic condition, our analysis reveals that a cleft surgical mission that involves local government and cleft foundation collaboration is paramount to tackling the sociodemographic challenge, especially in the rural- archipelago settings. A cleft surgical mission in this context is a time-limited medical outreach program offering complimentary surgical treatment for CL/P in areas lacking healthcare services. Teams of volunteers comprising oral and maxillofacial surgeons, plastic surgeons, anesthesiologists, nurses, and other medical professionals went to distant or low-income regions of Indonesia. They aim to conduct cleft operations and offer comprehensive care for individuals with cleft lips or palates.

Collaboration between healthcare providers, government agencies, local communities to engage with parents, and non-profit organizations is crucial to establishing sustainable healthcare systems that cater to cleft patients’ specific needs in urban and rural areas.^21^ These sociodemographic conditions highlight the need for tailored healthcare planning, resource allocation, infrastructure development, and health promotion strategies to address the CL/P healthcare needs of urban and rural populations, respectively.

The current study indicated that CL/P cases are less of a concern to regional policymakers because CL/P is not a predominant illness. Our conversation with one of the hospital employees shows that CL/P is a non-communicable disease, so the government often overlooks it. Including budget allocation for hospitals, due to limited funds, the procurement of tools to support the management of CL/P is not a priority. Cleft foundations, such as Celebes Cleft Center (CCC)^1^, have been established to help reduce the burden on referral hospitals. CCC's social work has greatly assisted in delivering biomedical treatments to various remote areas, particularly in eastern Indonesia, which lacks medical specialists who can treat patients and adequate equipment and facilities. According to the patient report, CCC provided free surgery to 250 patients in 2019.^22^ In addition to performing surgery, assisting in the provision of prostheses, and providing therapy, we suggest that CCC should also address the social problems associated with CL/P, particularly the lack of parental understanding of the treatments and therapy, as well as the societal effects that the patient may experience if not treated comprehensively. CCC must also encourage parents and the community to provide moral guidance. How parents in Makassar enable exposure to various experiences and access to education can serve as educational materials for CCC. If CCC cannot address these social issues during the social mission, the medical team at the regional general hospital can participate by educating the public, particularly parents of CL/P patients, so that comprehensive, timely, and multidisciplinary medical treatments can be conducted.

The strength of this ethnographic study is the researchers’ thorough, qualitative approach to exploring the subtle, contextual elements impacting cleft patients’ lived experiences in urban Makassar and rural Selayar Islands, Indonesia. The study uncovered extensive, first-hand experiences of cleft treatment access, community perspectives, and individual self-perceptions through in-depth interviews and immersion observations. The tiny, regionally-focused sample may only partially depict Indonesia's societal context and generational transitions in cleft patient experiences. Qualitative data gives valuable insights, but the researchers’ fieldwork duration and self-reported and observational data's subjectivity may limit the conclusions. Future research could expand the geographic and demographic scope, use longitudinal methods, and triangulate qualitative and quantitative findings to strengthen the empirical foundation for addressing complex sociodemographic challenges in cleft care management in Indonesia and similar contexts.

## Conclusion

CL/P management disparities in access and care exist between rural and urban areas. Socio-demographic factors play a crucial role, where education levels among urban parents positively impact treatment-seeking behaviors. Policymakers and community interventions are needed to address this equity, with targeted policies and initiatives to improve healthcare access in rural areas and establish specialized cleft centers. Ongoing efforts, such as collaborative initiatives and surgical missions, help to overcome sociodemographic challenges and promote comprehensive management.

## Notes

1. **CCC** is a cleft foundation based in Makassar. It provides free surgical treatments and education for cleft patients in the eastern part of Indonesia.

## Appendix

**List of abbreviations:**
CL/P: Cleft Lip and/or Palate
CCC: Celebes Cleft Center
UNHAS: Universitas Hasanuddin (Hasanuddin University)
PUSKESMAS: *Pusat Kesehatan Masyarakat* (Community Health Center)
